# Interferon-γ inhibits interleukin-1β-induced matrix metalloproteinase production by synovial fibroblasts and protects articular cartilage in early arthritis

**DOI:** 10.1186/ar2960

**Published:** 2010-03-22

**Authors:** Charlotte E Page, Shaun Smale, Sara M Carty, Nicholas Amos, Sarah N Lauder, Rhian M Goodfellow, Peter J Richards, Simon A Jones, Nicholas Topley, Anwen S Williams

**Affiliations:** 1 Section of Rheumatology, University Hospital of Wales, Cardiff and Vale NHS Trust; Heath Park, Cardiff, Wales, CF14 4XW, UK; 2 Section of Rheumatology, Department of Medicine, School of Medicine, Cardiff University, Heath Park, Cardiff, Wales, CF14 4XN, UK; 3 Department of Infection, Immunity & Biochemistry, School of Medicine, Cardiff University, Heath Park, Cardiff, Wales, CF14 4XN, UK

## Abstract

**Introduction:**

The first few months after symptom onset represents a pathologically distinct phase in rheumatoid arthritis (RA). We used relevant experimental models to define the pathological role of interferon-γ (IFN-γ) during early inflammatory arthritis.

**Methods:**

We studied IFN-γ's capacity to modulate interleukin-1β (IL-1β) induced degenerative responses using RA fibroblast-like synoviocytes (FLS), a bovine articular cartilage explant (BACE)/RA-FLS co-culture model and an experimental inflammatory arthritis model (murine antigen-induced arthritis (AIA)).

**Results:**

IFN-γ modulated IL-1β driven matrix metalloproteinases (MMP) synthesis resulting in the down-regulation of MMP-1 and MMP-3 production *in vitro*. IFN-γ did not affect IL-1β induced tissue inhibitor of metalloproteinase-1 (TIMP-1) production by RA FLS but skewed the MMP/TIMP-1 balance sufficiently to attenuate glycosaminoglycan-depletion in our BACE model. IFN-γ reduced IL-1β expression in the arthritic joint and prevented cartilage degeneration on Day 3 of AIA.

**Conclusions:**

Early therapeutic intervention with IFN-γ may be critical to orchestrate tissue-protective responses during inflammatory arthritis.

## Introduction

Interferon-γ (IFN-γ) is traditionally regarded as a proinflammatory cytokine by virtue of its strong macrophage-activating potential and its association with Th1 driven immune responses. This view is predominantly derived from *in vitro *observations at the cellular level. This belief has no doubt also contributed to our over-simplified understanding of major human autoimmune disorders such as rheumatoid arthritis (RA), multiple sclerosis and insulin-dependent diabetes mellitus and guided the development of therapeutic strategies for these diseases for over two decades. Accumulating contradictory findings from several *in vivo *experimental models of disease [1-7] as well as the suppressive role of IFN-γ upon interleukin-17 [8] signifies a need to rethink the doctrine of proinflammatory and disease-enforcing function for IFN-γ in autoimmune diseases such as RA.

The strong protective role for IFN-γ is exemplified in experimental models of arthritis, whereby genetic disruption of the IFN-γ receptor or IFN-γ results in increased disease activity [5-7]. In humans also a key immuno-modulatory function for IFN-γ may be postulated in very early arthritis. Raza et al demonstrated that patients presenting with inflammatory joint pain, joint related soft tissue swelling or early morning stiffness or a combination thereof for approximately three months displayed a distinct, but transient, synovial cytokine profile [9]. In the cohort of patients who went on to developed RA, a broad range of T-cell, macrophage and stromal cell related cytokines were elevated in synovial fluid samples; however, IFN-γ was never detected. If the cytokines present in the early rheumatoid lesion define the microenvironment which is required for RA pathology then, based upon current data from mice and humans, IFN-γ may prove to be a potentially important disease-modifying cytokine. The role of IFN-γ during the early phase of inflammatory arthritis has not been extensively studied.

In RA the first few months after symptom onset represents a pathologically distinct phase of disease which may translate into a therapeutic window and crucial target for implementing aggressive treatment protocols to permanently switch off or significantly arrest the disease process [9-11]. Detailed mechanistic information relating to the earliest stages of RA is limited by several contributory factors: (i) the changing clinical definition of early RA [12], (ii) access to sensitive imaging tools to detect early joint damage, (iii) traditional use of radiographic evaluation of bone erosion to quantify destructive pathology, (iv) cartilage degeneration (the earliest sign of joint damage in RA) is inherently problematic to visualise. Consequently, the role of IFN-γ in modulating articular cartilage during early RA has not been investigated previously.

The synovial membrane, recognised as the primary pathogenic site in RA, has been particularly informative in the study of novel therapeutic agents. Fibroblast-like synoviocytes (FLS), the major cellular constituent of the synovial membrane, are the main contributors to cartilage-degrading matrix metalloproteinase (MMP) over-production within the arthritic joint [13-15]. We used FLS cultured from RA synovial tissue specimens to model inflammation-induced MMP production and cartilage degeneration during the aetiopathogenesis of RA. Experimental data presented in this article, demonstrate an important regulatory function of IFN-γ in modulating FLS responses to IL-1β, specifically their ability to produce MMP-1 and MMP-3. This aspect of IFN-γ's functionality has not previously been reported. The balance between the bioactivities of MMPs and their inhibitors in the local tissue environment is a likely determinant of cartilage extracellular matrix degradation. We used bovine articular cartilage explants (BACE) to examine the biological activity of IL-1β activated RA FLS. Our results demonstrate the capacity of IL-1β activated RA FLS to degrade intact cartilage matrix and that IFN-γ potently protects articular cartilage against IL-1β induced damage *ex vivo*. In order to replicate the complexity of cellular and molecular interactions which regulate early cartilage injury in RA we utilised the well-published antigen-induced arthritis (AIA) model of inflammatory arthritis. Our data support a key protective role for IFN-γ signalling in limiting cartilage deterioration in early inflammatory arthritis; an outcome directed by IFN-γ's capacity to modulate the synovial expression of IL-1β and attenuate IL-1β-induced MMP production by FLS.

## Materials and methods

### Preparation and analysis of FLS

Human synovium was obtained from 13 consenting patients with RA who underwent synovectomy at the time of joint replacement (eight elbow replacements, and five knee replacements). At the time of joint replacement the mean age was 66 (range 46 to 77). Samples were obtained from nine females and four males; all patients had disease duration of greater than eight years. None of the patients had received anti-TNF therapy prior to surgery. Ethical approval for the study was obtained from Bro-Taf Health Authority (Reference 02/4692-Cardiff, Wales, UK). RA FLS were harvested and maintained in culture as previously described [7]. Briefly, RA FLS were harvested after collagenase digestion and expanded in culture flasks containing Dulbecco's modified Eagle's medium and Ham's F-12, at a 1:1 ratio, supplemented with 10% bovine fetal calf serum, antibiotics, L-glutamine, insulin and transferrin (DMEM/F12) (Life Technologies Invitrogen, Paisley, UK). RA FLS (n = 10) were used exclusively at the fourth passage. Experiments were performed in duplicate. RA FLS were stimulated with either recombinant human IL-1β (0.1 ng/ml), IFN-γ (0 to 10 ng/ml) or IL-1β in combination with escalating concentrations of IFN-γ (R&D Systems, Abingdon, UK). Control wells were incubated in DMEM/F12 only. Supernatants were harvested over a 72-hour timecourse and stored at -70°C prior to analysis. Cell viability was assessed by Alamar Blue Assay performed according to the manufacture's guidelines (Biosource International, Camarillo, California, USA) and was found to be greater than 95% at all time points.

### *Ex vivo *bovine articular cartilage explant (BACE) model

At fourth passage RA FLS (7.5 × 10^4^/well) were dispensed into a 12 well plate (NUNC™, Fisher Scientific, Loughborough, UK). Cells were grown until 90% confluent, normally within three days of passage. Full depth BACE were obtained from the metacarpal-phalangeal joint of immature (seven day old) bovine limbs using a published methodology [16]. Explants were maintained in DMEM/F12 for 24 hours at 37°C in a humidified incubator (5% CO_2_/air) prior to experimentation. Two plates were set up in parallel for each experiment, each condition was tested in duplicate and three RA-FLS cell lines were examined in all. On Plate 1: one BACE was dispensed into each of a 12-well plate. On Plate 2: one BACE was added to each well of a 12-well plate containing RA FLS. The BACE monocultures and RA FLS/BACE co-cultures were incubated for 72 hours with either DMEM/F12 or DMEM/F12 containing 0.1 ng/ml IL-1β, 10 ng/ml IFN-γ or the two cytokines in combination at the concentration specified. At end-point, supernatants were collected and stored at -70°C prior to analysis for MMP-3. BACE were also reserved, fixed in 10% neutral buffered formal saline, processed and then embedded in paraffin wax prior to histological evaluation. Proteoglycan depletion in fixed BACE was measured after Safranin-O/fast green staining.

### Enzyme-linked immunosorbant assay (ELISA)

Matched antibody pairs and protein standards for use in MMP-1 ELISA were obtained from R&D Systems (R&D Systems Europe, Ltd. Abingdon, UK). MMP-3 was measured using the specific human BioSource CytoSet™ CHC1544 ELISA kit (Life Technologies Corporation, Carlsbad, California, USA), MMP-13 using the human Quantikine^® ^DM1300 ELISA kit (R&D Systems Europe, Ltd. Abingdon, U.K) and TIMP-1 using the human DuoSet^® ^DY970 ELISA kit (R&D Systems Europe, Ltd. Abingdon, U.K).

Plasma cytokines (IL-1β and IFN-γ) were measured using mouse specific Quantikine^® ^Immunoassays (R&D Systems Europe, Ltd. Abingdon, UK). Assays were performed in accordance with the manufacturer's instructions.

### Mouse strains

Inbred wild-type (WT) BALB/c mice (IFN-γ+/+) were purchased from Charles River UK (Margate, UK). IFN-γ-deficient (IFN-γ-/-) mice on the BALB/c background were bred in house from breeding pairs provided by Professor Casey T Weaver (University of Alabama at Birmingham, AL, USA). All mice were maintained under barrier conditions and were pathogen free as assessed by regular microbiologic screening.

### Induction and assessment of murine AIA

Experiments were performed in seven- to eight-week-old male mice. Experimental procedures were performed in accordance with UK Home Office Project License PPL-30/1820 and 30/2361. For statistical analysis, a minimum of six animals per group were used; all experiments were duplicated to assure reproducibility of response. AIA was induced according to reported methodology [7,17]. Briefly, mice were immunized twice (one week apart) with an emulsion containing 1 mg/ml methylated bovine serum (mBSA) in complete Freund's adjuvant. Heat-inactivated Bordetella pertussis toxin was injected intraperitoneally as a non-specific immune activator (all reagents were from Sigma, Poole, UK). Twenty-one days after the initial immunization, arthritis was induced after an intra-articular (i.a.) injection of mBSA in PBS (0.1 μg/joint) into the right knee (stifle) joint.

Animals were evaluated daily for three days following i.a. mBSA injection. Arthritis was assessed by measuring knee joint diameters using a digital micrometer. The difference in joint diameter between the arthritic (right) and non-arthritic control (left) stifle joints in each animal gave a quantitative measure of swelling (in mm).

### Histologic assessment

Animals were sacrificed three days after i.a. mBSA injection. Knee joints were dissected, fixed in 10% neutral buffered formal saline, and decalcified with formic acid at 4°C prior to embedding in paraffin. Mid-sagittal serial sections (5 μm thickness) were cut and stained with haematoxylin and eosin (H&E) for visualising gross pathology or Safranin O-fast green staining for glycosaminoglycan (GAG) [18].

Disease severity was graded histologically by two blinded observers using defined disease activity parameters. Synovial hyperplasia (pannus formation) and cellular exudate were each scored from 0 (normal) to 3 (severe) whilst the synovial infiltrate was scored from 0 to 5. All parameters were subsequently summed to give an arthritis index (AI; expressed as the Mean ± SEM).

Cartilage depth and GAG depletion was measured (μm) in five random fields on the femoral articular surface. These areas were remote from the inflamed synovium.

### Immunohistochemistry

Paraffin wax-embedded knee sections were prepared and stained as previously described. Briefly, sections were treated with trypsin (0.1% w/v). Endogenous peroxidase and biotin were blocked using Chem-Mate Peroxidase blocking solution and biotin-blocking system respectively (Dako, Ely, UK). Sections were stained with rabbit anti-mouse IL-1β (1:50; Santa Cruz Biotechnology, Santa Cruz, CA, USA), and antibody binding was detected using the appropriate biotin-conjugated secondary antibody followed by StreptABComplex (Dako, Ely, UK). Control slides were probed with naive rabbit IgG and processed as above. Sections were developed using diaminobenzidine substrate and counterstained with haematoxylin.

### Statistical analysis

Tukey's test was used in conjunction with a one-way analysis of variance to analyze differences in cytokine and protease levels measured by ELISA. In AIA knee swelling was evaluated daily for three days following i.a. mBSA injection. Statistical analysis was performed using unpaired one-way analysis of variance. Disease severity in these animals was graded histologically using defined disease activity parameters. The Mann-Whitney U test was used to analyze differences between WT and IFN-γ-/- mice. Quantitative differences in GAG depletion (*ex vivo *and *in vivo*) were evaluated by a one-way analysis of variance. *P *values less than 0.05 were considered statistically significant. Values are expressed as the mean ± SEM.

## Results

### IFN-γ inhibits IL-1β induced MMP secretion by RA FLS

In RA cartilage damage is predominantly mediated by the MMPs; most notably MMP-1, -13 and -3 [19]. Their synthesis is driven by pro-inflammatory cytokines, most particularly IL-1 [20]. Collectively these MMPs degrade the major components of the extracellular matrix in cartilage and in bone. They are predominantly synthesised by both macrophages and FLS in the hyperplasic synovium. We studied IFN-γ's capacity to modulate IL-1β-induced MMP production by RA FLS.

**Figure 1:**
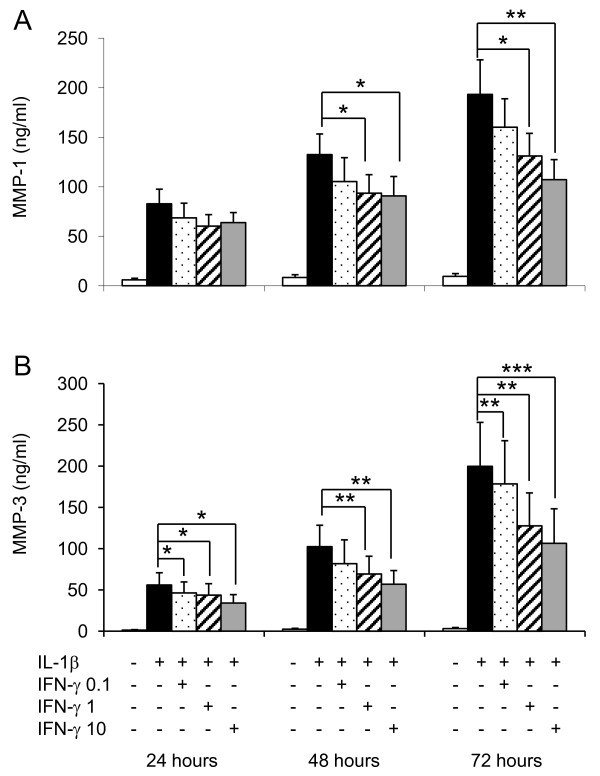
**IFN-γinhibits IL-1β induced MMP-1 and MMP-3 production by RA FLS *in vitro***. Fibroblast-like synoviocytes (FLS) were isolated from rheumatoid arthritis (RA) synovial tissue specimens. Ten RA FLS cell lines were studied in all. At fourth passage RA FLS were incubated with either culture medium, IL-1β (0.1 ng/ml), escalating doses of IFN-γ (0.1 to 10 ng/ml) or combinations of the two cytokines for 72 hours. Culture supernatants were harvested at 24, 48 and 72 hours. Protease concentrations were measured by ELISA; (A) MMP-1 and (B) MMP-3 (mean ± SEM) values are reported. Tukey's test was used in conjunction with a one-way analysis of variance to analyze differences in protease levels. *P *values less than 0.05 were considered statistically significant; * *P *< 0.05, **P < 0.01 and ***P < 0.001.

IFN-γ did not induce significant MMP-1 nor did it induce significant MMP-3 production (mean ± SEM, ng/ml) in RA FLS over 72 hours (IFN-γ (10 ng/ml) versus control media at 72 hrs; MMP-1 = 15 ± 6 vs. 9.5 ± 3 and MMP-3 = 3.7 ± 1.7 vs. 3.1 vs. 1.4). In contrast MMP-1 and MMP-3 expression increased in RA FLS in response to IL-1β activation (0.1 ng/ml) for periods up to 72 hours (Figure 1). Co-administration of IL-1β with increasing concentrations of IFN-γ resulted in a significant reduction in detectable MMP-1 and MMP-3 levels when compared to that elicited by the IL-1β alone (Figure 1).

By virtue of its capacity to metabolise type II collagen over interstitial type I and III collagens, MMP-13 is also implicated in the degradation of extracellular matrix within articular cartilage. In our studies we observed maximal MMP-13 production (mean ± SEM) in RA FLS 24 hours after stimulation with IL-1β (1.0 ± 0.3 ng/ml); which plateaued between 48 and 72 hours (0.8 ± 0.3 ng/ml). Co-stimulation with IFN-γ reduced MMP-13 secretion to 0.8 ± 0.1 ng/ml and 0.4 ± 0.08 ng/ml at 24 and 72 hours respectively. This reduction in MMP-13 production was not statistically significant. Of note, however, RA FLS secreted >100 fold more MMP-1 than MMP-13 under the same *in vitro *conditions, implying that perhaps production of MMP-1 by FLS is more important than that of MMP-13 in catalysing cartilage degradation in RA. IFN-γ is therefore able to negatively regulate pathological MMP secretion by RA FLS in a pro-inflammatory setting.

### IFN-γ does not affect tissue protective TIMP-1 secretion by RA FLS

**Figure 2:**
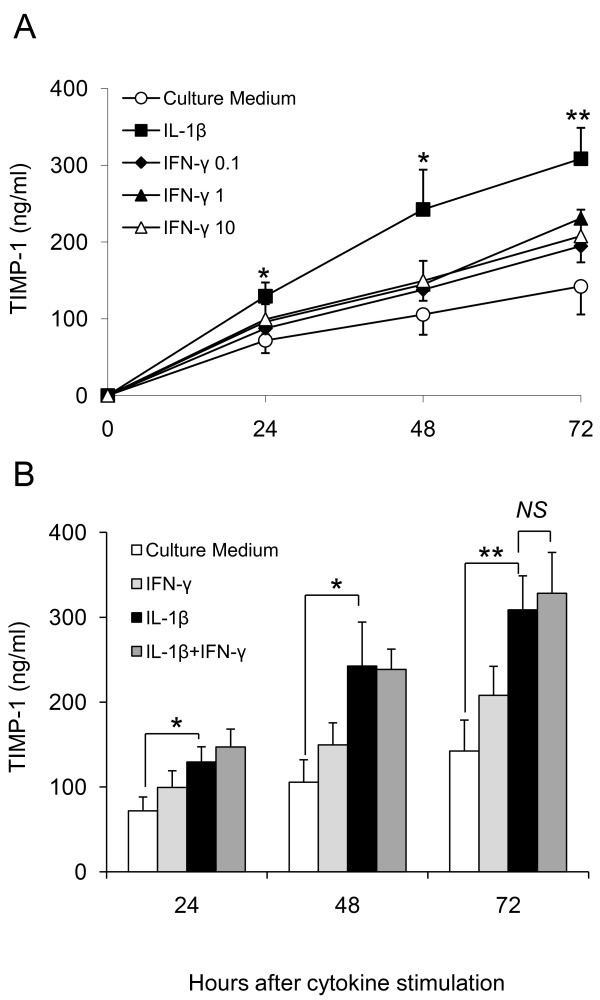
**IFN-γ does not affect tissue protective TIMP-1 secretion by RA FLS *in vitro***. Fibroblast-like synoviocytes (FLS) were cultured from synovial tissue specimens obtained from rheumatoid arthritis (RA) patients at synovectomy or joint replacement. Eight cell lines were used at fourth passage. Cells were incubated in culture medium or in medium containing escalating doses of IFN-γ (0.1 to 10 ng/ml), IL-1β (0.1 ng/ml) or combinations of the two cytokines for 72 hours. TIMP-1 levels were quantified by ELISA in culture supernatants at 24, 48 and 72 hours; mean ± SEM TIMP-1 concentrations are reported. **(A) **TIMP-1 levels were significantly increased by IL-1β but not escalating concentrations of IFN-γ. IFN-γ did not affect IL-1β induced TIMP-1 production by RA FLS; **(B) **represents data obtained for 10 ng/ml IFN-γ ± IL-1β (0.1 ng/ml). Tukey's test was used in conjunction with a one-way analysis of variance to analyze differences in TIMP-1 levels. *P *values less than 0.05 were considered statistically significant. *NS *denotes non-significant change, *P < 0.05 and **P < 0.005.

The enzymatic activity of the MMPs is strictly regulated by specific inhibitors, the tissue inhibitors of metalloproteinases family (TIMP). Since synovial lining cells in RA specifically overproduce TIMP-1 [19,21], the effect of IFN-γ on the generation of this important regulator of cartilage and extracellular matrix degradation was determined. RA FLS constitutively produced high levels of TIMP-1 in culture media without cytokine activation (Figure 2A). IFN-γ treatment only marginally increased TIMP-1 secretion (*P *= NS). Incubation of RA FLS with IL-1β for 72 hours resulted in a time dependent increase in the secreted levels of TIMP-1. Co-administration of IL-1β with concentrations of IFN-γ up to 10 ng/ml did not significantly alter TIMP-1 generation (Figure 2B).

### IFN-γ protects articular cartilage from IL-1β induced GAG depletion

**Figure 3:**
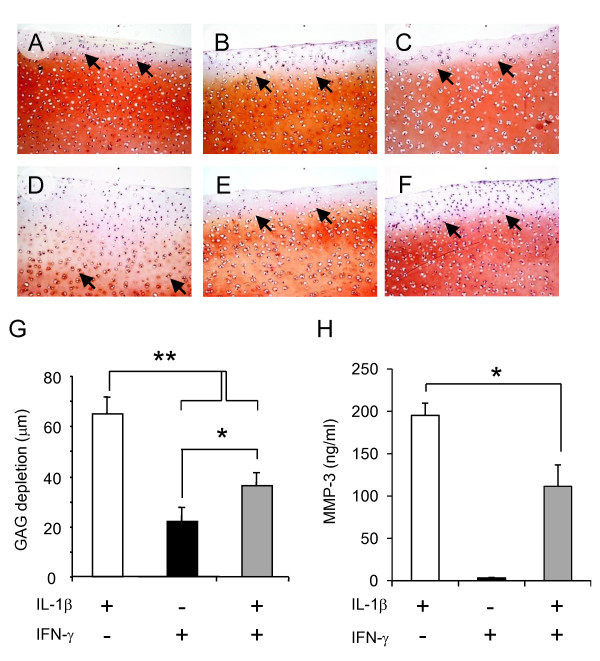
**IFN-γ protects articular cartilage from GAG depletion mediated by IL-1β activated RA FLS *ex vivo***. Full depth articular cartilage explants were obtained from bovine limbs (BACE). Two 12-well plates were set up in parallel, one with RA FLS the second without RA FLS. At the beginning of each experiment, one BACE was place in each well. BACE (± RA FLS) were incubated in DMEM/F12 or medium containing; 0.1 ng/ml IL-1β, 10 ng/ml IFN-γ or IL-1β with IFN-γ for 72 hours when cartilage explants and supernatants were reserved. Three RA FLS cell lines were tested; cytokines (alone or in combination) were examined in duplicate. Cartilage depletion at end point was visualised in Safranin-O/Fast Green-stained sections. Representative images from one experiment are reported **(A-F)**, reduced intensity of red stain denotes proteoglycan loss (original magnification × 20). A-C BACE cultured without RA FLS, D-E corresponding BACE from RA FLS co-cultures which had been incubated in DMEM/F12 medium containing: 0.1 ng/ml IL-1β (A and D), 10 ng/ml IFN-γ (B and E) or IL-1β with IFN-γ (C and F) for 72 hours. The depth of GAG depletion (μm) in each BACE, measured from the articular surface to the red/orange tidemark (denoted by black block arrow) is presented graphically in **(G)**; mean ± SEM for three experiments reported. MMP-3 was measured by ELISA in culture supernatants harvested from each well after 72 hours in culture. **(H) **MMP-3 (ng/ml); mean ± SEM for three experiments. One-way analysis of variance was used to analyze differences in GAG depletion and MMP-3 levels: *P < 0.05 and **P < 0.0001.

The balance between the bioactivities of MMPs and TIMPs in the local tissue environment is a likely determinant of cartilage extracellular matrix degradation. We therefore examined whether the sum of the biological activities of MMP/TIMP generated by IL-1β activated RA FLS was sufficiently skewed as to induce GAG depletion in a BACE model. After 72 hours in culture we noted diminutive reductions in Safranin-O/fast green staining in BACE incubated in DMEM/F12 or medium containing IL-1β IFN-γ or the two cytokines in combination (Figure 3A-C). GAG depletion, which penetrated the BACE by approximately 25 μm, was comparable in all BACE test culture conditions. This basal GAG depletion was attributed to our choice of media which was optimised for FLS rather than chondrocyte culture.

Three RA FLS cell lines were tested in all. In each case extensive GAG depletion (mean ± SEM) was observed in IL-1β activated BACE/RA FLS co-cultures (64 ± 7 μm, Figure 3D, G). This FLS dependent GAG depletion was significantly greater than the GAG depletion measured in BACE in the absence of RA FLS (29.7 ± 4.8 μm, Figure 3A; *P *< 0.001). Co-administration of IFN-γ potently protected the BACE from GAG depletion induced by IL-1β-activated RA FLS (36.2 ± 5.5 μm, Figure 3G); evidenced by the intense red staining for Safranin O (Figure 3F).

In order to determine whether IFN-γ safeguarded articular cartilage by modulating IL-1β induced RA FLS protease production we measured MMP-3 levels in supernatant at the 72-hour endpoint for our experiments. Consistent with our finding in RA FLS monocultures, we found that BACE/RA FLS co-cultures produced negligible MMP-3 in response to IFN-γ activation (3.1 ± 1.0 ng/ml). Co-administration of IL-1β with IFN-γ resulted in a significant reduction in detectable MMP-3 levels when compared to that elicited by IL-1β alone (Figure 3H). Our data imply that IFN-γ safeguards articular cartilage by modulating IL-1β induced protease production by RA FLS.

### Joint pathology in IFN-γ-deficient mice with early AIA

Comparison of the histopathology of synovial tissue during early RA, established RA, and in non-RA synovitis has shown subtle, but potentially important differences in histologic features, cytokine and protease expression patterns [22,23]. The histopathology of the early RA demonstrates mild proliferation of synovial lining cells, an abundant occurrence of mononuclear phagocytes (sublining layer) and a diffuse T cell infiltrate (subsynovial layer) [24]. Similar characteristic pathological features are evident in AIA during the acute phase, up to three days after arthritis induction [7,25,26]. We therefore elected to run our *in vivo *experiments for three days; a period of time which we felt best represented very early RA.

**Figure 4:**
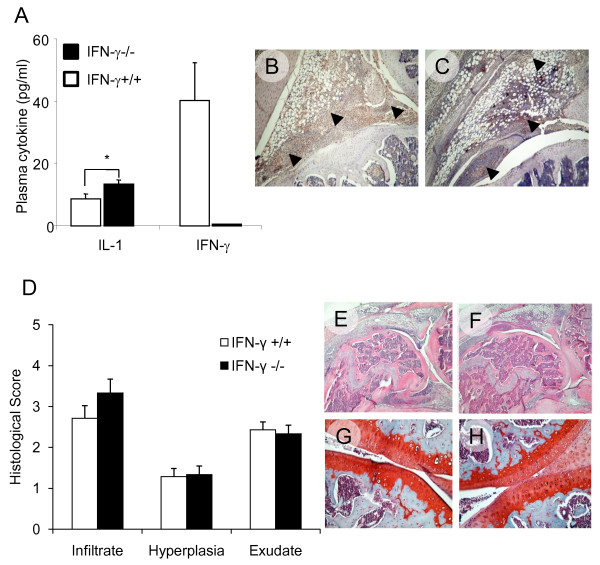
**Development and progression of early arthritis in IFN-γ deficient mice**. Mono-articular antigen-induced arthritis (AIA) was triggered in mice previously immunized against methylated bovine serum albumin (mBSA); arthritis was induced after an intra-articular (i.a.) injection of mBSA. Plasma and synovial cytokine expression was assessed together with histological measures of arthritis severity during early arthritis (one day after i.a. mBSA injection). **(A) **Plasma cytokine expression (mean ± SEM; pg/ml) shown in wild-type and IFN-γ-/- mice one day after AIA induction; *P < 0.05. **(B) **and **(C)**, representative immunohistochemical staining for IL-1β in joint sections from IFN-γ+/+ and IFN-γ-/- mice respectively; intense positive brown staining (denoted by black block arrows) present in the synovial lining layer, joint exudate and focal areas of inflamed synovium, (original magnification ×10). Arthritis severity was graded in histological sections from IFN-γ+/+ (n = 7) and age matched IFN-γ-/- (n = 6) mice one day post arthritis induction. **(D)**. Individual parameters of arthritis severity (mean ± SEM) reported. **(E)**. Representative H&E stained para-sagittal section demonstrating histopathology of a IFN-γ+/+ knee joint processed one day post arthritis induction. **(F)**. Corresponding section from IFN-γ-/-, showing comparable cellular infiltration, synovial hyperplasia and joint exudate (original magnification ×4). **(G) **and **(H**). Representative, Safranin-O/Fast green stained, para-sagittal section demonstrating typical cartilage architecture in arthritic knees on Day 1. Replete GAG staining (red) in articular cartilage; **(G)** IFN-γ+/+ and **(H)** IFN-γ-/- (original magnification ×20).

Wild type (IFN-γ+/+) and IFN-deficient (IFN-γ-/-) mice were both highly susceptible to the development of AIA. One day after intra-articular (i.a.) administration of methylated bovine serum albumin (mBSA) we detected small but significant (*P *< 0.05) increases in plasma levels of IL-1β in IFN-γ-/- mice (13.4 ± 1.1 pg/ml vs. 8.7 ± 1.3 pg/ml for WT). IFN-γ levels were only detectable in WT mice (36.8 ± 0.4 pg/ml) (Figure 4A). IL-1β nor IFN-γ were detectable in plasma samples at subsequent time points (Day 2 and Day 3). We observed moderate IL-1β expression in joints affected by AIA on Day 1. Abundant staining was detected in the synovial lining layer, joint exudate and focal areas of synovial infiltration; it was comparable in WT and IFN-γ-/- mice (Figures 4B, C). Histological evaluation of joint tissue specimens demonstrated that all inflammatory parameters were equivalent; this was reflected in the Arthritis Index (AI) which was not significantly different in WT and IFN-γ-/-; 6.4 ± 0.4 vs. 7.0 ± 0.6 (Figure 4D). There was no evidence of cartilage depletion by Safranin-O/fast green staining despite the discernible inflammatory synovial environment (Figure 4E-H).

### IFN-γ protects the joint against early articular cartilage damage in AIA

**Figure 5:**
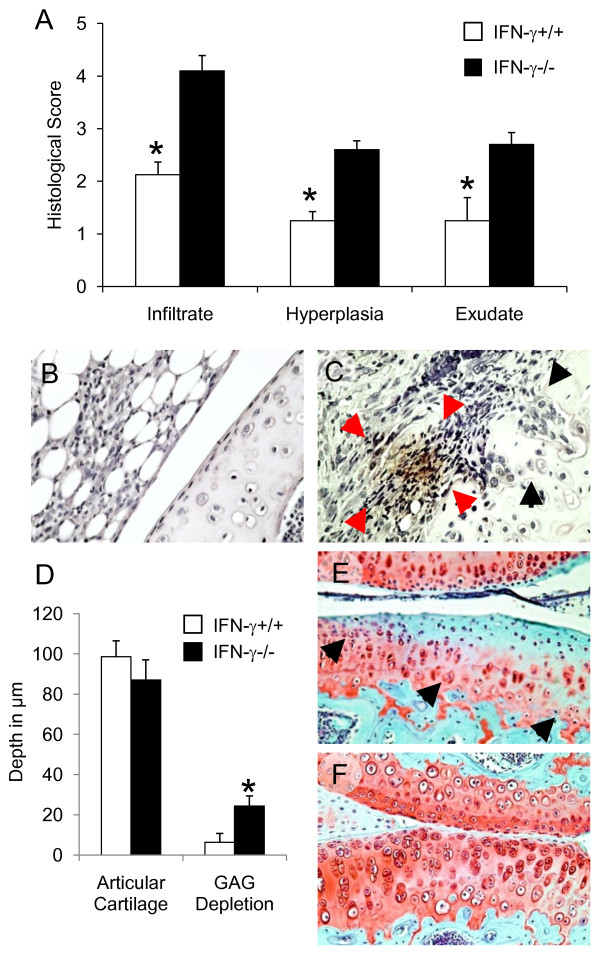
**IFN-γ protects the synovial joint from degenerative articular changes in early experimental arthritis**. Mono-articular antigen-induced arthritis (AIA) was triggered in mice previously immunized against methylated bovine serum albumin (mBSA); arthritis was induced after an intra-articular (i.a.) injection of mBSA. Changes in joint architecture were measured histologically in joint tissue sections from IFN-γ+/+ (n = 8) and age matched IFN-γ-/- (n = 10) three days after AIA induction. **(A)**. Individual parameters of arthritis severity were determined from H&E stained arthritic knee joints; (mean ± SEM) reported; *P < 0.01. IL-1β expression was visualised in arthritic joints excised three days post AIA induction using cytokine specific immunohistochemistry. **(B) **and **(**C)****. Representative paraffin wax sections from IFN-γ+/+ and IFN-γ-/- stained for IL-1β (original magnification ×40). Negligible IL-1β staining observed in synovial tissues from IFN-γ+/+ mice (B). In IFN-γ-/- mice (C) intense IL-1β staining often seen in the pannus (denoted by red block arrows); articular cartilage frequently found damaged with surface fibrillation and clefts extending into transitional zone (denoted by black block arrows). Serial sections were stained with Safranin-O/Fast green. Cartilage depth and the depth of GAG depletion were calculated for each mouse from an average of five fields per femoral head. **(D)**. Mean ± SEM depths in μm reported for IFN-γ+/+ and IFN-γ-/- (*P < 0.01). **(**E)****. Representative Safranin-O/Fast green stained para-sagittal section from IFN-γ-/- demonstrating severe GAG depletion (block arrows) and reduced cellularity. **(F)**. Corresponding section from a IFN-γ+/+ knee joint, minimal GAG depletion observed in the cartilage on the articular surfaces (original magnification ×20).

By Day 3 all parameters of early arthritis activity were significantly higher in IFN-γ-/- mice (AI = 9.4 ± 0.5) compared with their IFN-γ sufficient wild-type (AI = 4.6 ± 0.5) counterparts (Figure 5A). The leukocytes recruited to the inflamed joint during this phase provide a cellular source for one of the most prominent catabolic factors that compromises cartilage integrity, the pro-inflammatory cytokine IL-1 [20]. We found negligible IL-1β staining in the synovial tissues from WT mice on Day 3 (Figure 5B). In contrast, IFN-γ-/- mice exhibited strong expression of IL-1β immuno-reactivity both in the patches of infiltrating cells and in the inflamed synovial lining layer, most particularly in the pannus adjacent to areas of cartilage damage (Figure 5C). We did not observe any chondrocyte-associated IL-1β expression in WT or IFN-γ-/- mice at any of the time points studied. Control slides were probed with naive rabbit IgG in all staining runs, non-specific staining was never detected (data not shown).

Finally, we questioned whether IFN-γ ablation precipitated cartilage injury during early inflammatory arthritis. AIA of three-day duration was sufficient to trigger widespread articular damage (visualised by diminished Safranin-O staining) in the articular cartilage of IFN-γ-/- mice (Figure 5D, E). Examination of joints from WT mice revealed almost normal structural integrity (Figure 5F).

## Discussion

It is likely that the net effect of IFN-γ *in vivo *is reliant upon the context in which it is presented; cell/cytokine milieu, site of action and disease. We used a series of *in vitro *and *in vivo *approaches to demonstrate that IFN-γ potently protected articular cartilage from inflammation-induced degradation. Studies outlined herein reveal a close link between IFN-γ and the control of IL-1β-induced articular cartilage damage during a critical phase in the aetiopathogenesis of inflammatory arthritis. Our findings highlight a potentially important disease-modifying functionality for IFN-γ in early arthritis which might signify a rethink with regard to timely intervention with IFN-γ in innovative treatment protocols for inflammatory joint diseases such as RA.

To define the manner by which IFN-γ might regulate catabolic responses induced by IL-1β in humans we utilised an *in vitro *model system using the structural cells that constitute the inflammatory pannus, namely, FLS cultured from RA synovial tissue specimens. RA FLS, which were originally thought to be relatively inert structural components of the tissue, are now known to play an important role in regulating tissue homeostasis in healthy and diseased joints [27,28]. Our observations confirm IL-1β as a potent activator of MMP-1 and MMP-3 production by FLS. However co-incubation with IFN-γ, which does not itself induce MMP-1 or MMP-3, significantly reduced IL-1-induced MMP-1 and MMP-3 production. MMP-1 and MMP-3 are abundant in human RA tissues, sera and synovial fluids and may be indicative of poor prognosis and progressive destructive disease [21,29-31]. Our data suggest an important role for IFN-γ in regulating MMP production by RA FLS. IFN-γ is thus capable of modulating one of the most important processes driving tissue degeneration in arthritis.

The mechanism by which IFN-γ regulates IL-1β-induced MMP production by RA FLS is not known. It remains to be seen whether IFN-γ modulates RA FLS responsiveness to IL-1β by modulating cellular expression of IL-1 receptor and/or IL-1 receptor antagonist (IL-1RA) and/or regulating signal transducer and activator of transcription dependent signalling through nuclear factor κB.

Extracellular matrix degradation in arthritis may be driven by MMPs but their capacity to alter tissue architecture is tightly regulated by their endogenous inhibitors; the TIMP family [32]. We observed that treatment of FLS with IL-1β caused significant induction of TIMP-1. Co-administration of IFN-γ, however, had no significant modulating effect. The relationship between elevated TIMP-1 levels in RA patients and radiographic progression is not entirely clear. A recent study demonstrated that serum TIMP-1 levels were significantly higher in patients with progressive RA over those who had radiographically stable disease [19]. These findings differed from previous studies, in which no association was observed between TIMP-1 levels and radiographic outcome [13,33,34]. In the context of our *in vitro *studies MMP-1, MMP-3 and TIMP-1 levels were elevated in response to IL-1β treatment. Since both destructive and protective enzymes were induced, it is conceivable that the ratio of MMP:TIMP should be considered as an indicator of activity when predicting outcome in terms of IFN-γ's regulatory potential. To explore this we used a BACE/RA FLS co-culture model to test whether the balance of enzymes and inhibitors induced by co-administration IL-1β with IFN-γ was sufficient to shield cartilage from IL-1β induced damage. An average MMP-3:TIMP-1 ratio of 0.65 and MMP-1:TIMP-1 ratio of 0.62 were calculated for RA FLS activated by IL-1β alone. These ratios were reduced by approximately 50%, to 0.33 and 0.34 respectively, following the co-administration of IFN-γ. This decrease in MMP:TIMP ratio was sufficient to elicit a strong net protective effect upon the cartilage explants, thereby identifying an additional homeostatic role for IFN-γ in limiting inflammation-induced cartilage damage.

We found that IFN-γ gene ablation made mice more susceptible to inflammation-induced cartilage injury. Using an experimental model of early arthritis we discovered that IFN-γ-/- mice exhibited detrimental, often irreversible, phenotypic changes in articular cartilage structure. The extensive GAG depletion and frequent lesions observed in IFN-γ-/- mice were not seen in WT animals.

IL-1β is considered to be the principal cartilage-catabolic cytokine in arthritis. In murine AIA, significant and temporal changes in cytokines (IL-1β, TNFα, IFN-γ, IL-6) and factors that govern extracellular matrix turnover (MMP-3, MMP-13, TIMP-1) occur during the acute phase, Days 1 to 3 after arthritis induction [25,35,36]. In line with published information, the current data demonstrate maximal IL-1β expression in the joint on Day 1 which is also when peak IFN-γ levels are detected. By Day 3, IFN-γ-/- mice demonstrated elevated IL-1β expression levels compared to WT animals in the intimal synovial lining layer and most particularly in the pannus adjacent to areas of damaged cartilage. The prolonged expression of IL-1β within the joint and the sustained activation of synovial cells by IL-1β in the absence of IFN-γ are likely to aggravate cartilage damage in AIA.

The precise mechanism by which IFN-γ helps maintain the structural integrity of the joint is ill-defined but is undoubtedly multi-factorial. We have previously demonstrated that IFN-γ elicited potent negative-regulatory function on neutrophil recruitment in arthritis [7]. These cells, usually found in abundance in patchy synovial infiltrates, in patients with very early RA [23,24,37], secrete tissue degrading proteases. Furthermore, IFN-γ acted synergistically with IL-1 to stimulate IL-1RA production and inhibit MMP-3 and MMP-13 release in human chondrocytes [38,39]. IFN-γ also inhibits MMP-3 mRNA expression in IL-1 induced primary human macrophages [40]. IFN-γ is therefore able to curtail IL-1β induced MMP production by many cell types implicated in perpetuating structural damage in arthritis; cellular responses that could potentially dampen early tissue destruction and joint damage.

Accumulating data reveal diverse mechanisms by which IFN-γ antagonizes inflammatory and/or pathological pathways [41]. In arthritis, for example, IFN-γ: (i) inhibits neutrophil trafficking [7], (ii) promotes regulatory T-cell function [42], (iii) suppresses Th17 cell expansion [43], thereby attenuating inflammation and preventing T-cell driven osteoclast activation and bone erosion [44] and (iv) limits the biological activity of interleukin-18 [45]. Our data provide additional unifying evidence for IFN-γ's protective function in arthritis: IFN-γ lowered IL-1β-induced MMP production by RA FLS, altered the MMP/TIMP balance in favour of cartilage protection *ex vivo*, modulated synovial IL-1β expression in AIA and ameliorated early cartilage injury *in vivo*. These data advance our understanding of the immuno-protective function of IFN-γ within the synovial joint and go some way towards identifying mechanisms by which IFN-γ regulates a chondro-protective outcome during early inflammatory arthritis.

## Conclusions

IFN-γ was previously advocated as safe and well tolerated for the treatment of chronic progressive RA and juvenile chronic arthritis [46,47]. The multicenter, randomized, double-blind trial by Veys et al [46] found that recombinant IFN-γ proved no more effective than placebo in patients with chronic progressive RA. However, the management of RA has changed considerably over the past decade with recognition that earlier treatment provides superior outcomes. It is possible that an early intervention with IFN-γ may lead to a different result. If a *therapeutic window *exists for RA, then our studies and those of other groups support a role for IFN-γ in the biological armamentarium against RA.

## Abbreviations

AI: arthritis index; AIA: murine antigen-induced arthritis; BACE: bovine articular cartilage explant; FLS: fibroblast-like synoviocytes; GAG: glycosaminoglycan; H&E: haematoxylin and eosin; i.a.: intra-articular; IFN-γ: Interferon-γ; IL-1β: interleukin-1β; mBSA: methylated bovine serum; MMP: matrix metalloproteinases; RA: rheumatoid arthritis; TIMP-1: tissue inhibitor of metalloproteinase-1.

## Competing interests

The authors declare that they have no competing interests.

## Authors' contributions

CEP carried out the immunoassays, harvested and cultured RA FLS, performed *ex vivo *BACE/FLS studies and helped to draft the manuscript. SS, SMC, NA and SNL carried out the immunoassays, and harvested and cultured RA FLS. RMG arranged the ethical approval for tissue collection and helped to draft the manuscript. PJR carried out the immunohistochemical staining. NT and SJ were involved in revising the manuscript and providing critiques for important intellectual content. ASW conceived of the study, performed the *in vivo *experiments, made substantial contributions to the experimental design and was involved in the acquisition, analysis and interpretation of data.
